# Satellite mapping exposes global inequities in lake water quality across 145 countries

**DOI:** 10.1093/nsr/nwag445

**Published:** 2026-07-20

**Authors:** Ming Shen, Tianci Qi, Juhua Luo, Zhigang Cao, Wei Zhi, Yunlin Zhang, Daniel A Maciel, Tiit Kutser, Anthony Gidudu, Zhe Sun, Steven Loiselle, Hongtao Duan

**Affiliations:** State Key Laboratory of Lake and Watershed Science for Water Security, Nanjing Institute of Geography and Limnology, Chinese Academy of Sciences, China; University of Chinese Academy of Sciences, China; State Key Laboratory of Lake and Watershed Science for Water Security, Nanjing Institute of Geography and Limnology, Chinese Academy of Sciences, China; State Key Laboratory of Lake and Watershed Science for Water Security, Nanjing Institute of Geography and Limnology, Chinese Academy of Sciences, China; University of Chinese Academy of Sciences, China; State Key Laboratory of Lake and Watershed Science for Water Security, Nanjing Institute of Geography and Limnology, Chinese Academy of Sciences, China; State Key Laboratory of Water Disaster Prevention, Yangtze Institute for Conservation and Development, Hohai University, China; State Key Laboratory of Lake and Watershed Science for Water Security, Nanjing Institute of Geography and Limnology, Chinese Academy of Sciences, China; University of Chinese Academy of Sciences, China; Instrumentation Lab for Aquatic Systems (LabISA), Earth Observation Coordination of National Institute for Space Research (INPE), Brazil; Estonian Marine Institute, University of Tartu, Estonia; Department of Geomatics and Land Management, Makerere University, Uganda; State Key Laboratory of Lake and Watershed Science for Water Security, Nanjing Institute of Geography and Limnology, Chinese Academy of Sciences, China; University of Chinese Academy of Sciences, China; Dipartimento di Biotecnologie, Chimica e Farmacia, CSGI, University of Siena, Italy; State Key Laboratory of Lake and Watershed Science for Water Security, Nanjing Institute of Geography and Limnology, Chinese Academy of Sciences, China; University of Chinese Academy of Sciences, China

## Abstract

For the first time, global lake water quality can be assessed consistently across countries. Satellitemachine learning assessment of 16,409 lakes across 145 countries reveals that only two out of every three lakes meet SDG water quality standards, exposing profound inequalities in freshwater security that affect billions of people.

Freshwater lakes serve as indispensable drinking water sources for billions of people worldwide. However, the 2025 UN SDG report revealed that existing UN monitoring systems failed to assess lake water quality in more than 60% of member states, mainly low- and middle-income countries in Africa, Asia, and South America (SDG indicator 6.3.2: “proportion of water bodies with good ambient water quality”) [1,2]. These monitoring gaps pose three major governance challenges: policy paralysis, as the lack of evidence hampers effective responses to the water quality crisis; climate vulnerability, because the growing risks of water scarcity driven by evaporation and extreme weather remain unquantified; and equity failure, which disproportionately affects regions least capable of conventional monitoring. This triad of issues exacerbates water insecurity in vulnerable areas, undermining global efforts toward sustainable development. Projections suggest that by 2030, the health and livelihoods of up to 4.8 billion people could be at risk if water quality and monitoring do not improve [1,3].

Exacerbating these shortcomings, conventional SDG 6.3.2 assessments depend on expensive *in situ* measurements of five physicochemical parameters [dissolved oxygen (DO), nutrients, pondus hydrogenii (pH), chemical oxygen demand (COD), and ammonia nitrogen (NH₄-N)]. However, the poorest half of the world contributes less than 3% of nearly two million reported water quality observations [1]. Although satellites can detect optically active water quality parameters (OWQPs)—such as chlorophyll-a, suspended particulate matter, colored dissolved organic matter, and Secchi disk depth [4]—critical SDG metrics like DO, nutrients, pH, COD, and NH₄-N lack direct optical signatures [5]. This technical gap creates a vicious cycle as the regions most in need of monitoring are those least covered [6].

**Two-stage random forest water quality assessment model.** To overcome these dual limitations, we developed GlakeWQ (Figs S1 and S2), an integrated satellite-machine learning framework that harmonizes 1.39 million Sentinel-2 MSI images (2019–22) with China’s national water quality standard (GB3838-2002) and enables the analysis of 16 409 lakes (≥10 km²) across 145 countries (Figs S3) [7]. Our two-stage data-driven approach first applied random-forest regression to convert spectral reflectance from six MSI bands (443, 492, 560, 665, 704, 740 nm) and five band ratios into OWQPs, achieving a mean absolute percentage error (MAPE) of ≤31.34% (Figs S4a–d and S5). Subsequently, a random-forest classification model was developed to establish the first direct linkage between satellite-derived OWQPs and SDG-compliant water quality classes. At this stage, OWQPs were mapped to physicochemical-based classes (Levels I–III = ‘good’; IV-inferior V = ‘poor’) following China’s GB3838-2002 standard (Table S1), which aligns with the United States (US), the European Union, and other countries’ benchmarks for DO, nutrients, and organic pollutants (Table S2). By adopting GB3838-2002 worldwide, we provide a unified, objective global benchmark. The water quality of centralized drinking water sources must fall into the good classes, ensuring they are suitable for consumption after conventional treatment. Model validation through 5-fold cross-validation demonstrated an overall classification accuracy of 75.4% across diverse global aquatic systems based on 10 957 *in situ* observations (Figs S4e, S6–S8, and Tables S3 and S4). Water bodies were classified as ‘good’ only when more than 80% of their surface area maintained stable good conditions for over 80% of the monitoring period, following UN SDG 6.3.2 reporting guidelines (Fig. S2) [8].

Two fundamental scientific advances emerge from our analysis:

**Global water quality disparities.** The satellite observations in this study indicated profound continental disparities, with only 63.3% of lakes

(10 385 out of 16 409) globally meeting ‘good’ water quality standards (levels I–III under GB3838-2002) (Fig. 1a). While Europe (73.1%) and North America (80.6%) showed relative water security, developing regions exhibited lower security—Asia (41.7%), South America (27.4%), Africa (20.3%), and Oceania (16.1%). Notably, the 70 countries unreported to the UN—mostly in Africa, Asia, and South America—had an average SDG 6.3.2 score of 44.3% ± 33.9%, about 25 points lower than the developed-country average (69.3% ± 27.3%). This spatial deficiency formed distinct ‘risk belts’ across equatorial Africa and South/Southeast Asia, where limited infrastructure hindered conventional monitoring.

**Figure 1.**
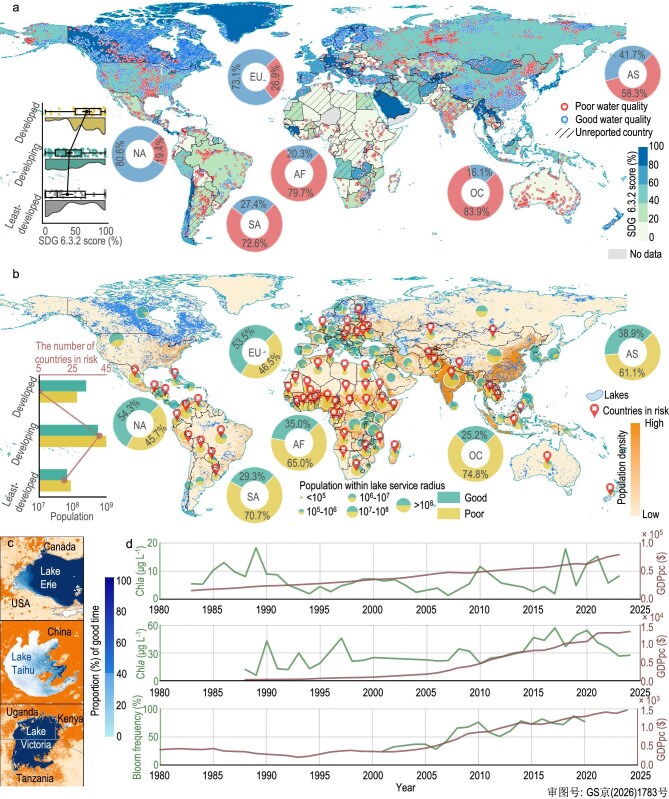
Global lake water quality, human exposure, and long-term trends. (a) Global status of lake water quality and national progress toward SDG 6.3.2. The panel includes global maps of lake water quality status. Box plots summarize water quality statistics categorized by country development level, and pie charts summarize the statistics by continent. Note that the unreported country indicated nations that did not provide official reports to the UN on lake water quality. (b) Population exposure to different lake water quality classes. The lake service radius is defined using a 30-km buffer, which accounts for feasible pipeline costs. Lakes located in endorheic basins, which may be naturally saline, were excluded from this analysis. (c) Examples of water quality patterns for representative lakes in countries of different development levels, including Lake Erie (western part, USA/Canada), Lake Taihu (China), and Lake Victoria (Uganda, Kenya, Tanzania). The base map shows population data sourced from the WorldPop project. (d) Long-term trends showing the relationship between lake health (measured by chlorophyll-a concentrations or algal bloom frequency) and per capita GDP for a selection of typical lakes.

**Population exposure crisis.** To align with human water supply requirements, we restricted this analysis to freshwater resources, excluding endorheic lakes due to salinity concerns. Of the 14 622 exorheic freshwater lakes identified, their service areas, defined as 30-km buffer zones that represent feasible water transfer distances, supported approximately 2 billion people. Notably, 57.4% of this population faced water scarcity due to poor water quality, reflecting an inequitable burden disproportionately borne by vulnerable populations (Fig. 1b). A total of 66 countries were at high risk, with more than 60% of their populations relying on freshwater lakes of poor water quality for drinking water. Developing and least-developed countries (63.7%) had exposure rates 1.8 times higher than those of developed economies (34.7%), as seen in high-risk hotspots such as India (79.8%), Indonesia (82.8%), Thailand (75.2%), and the African Great Lakes region (Uganda 65.8%, Tanzania 80.5%, Kenya 76.5%). This crisis forces either costly advanced treatment or dependence on contaminated alternatives, thereby exacerbating inequities. By contrast, more than 80% of people in developed economies such as Japan, South Korea, and Germany had access to good-quality lake water, demonstrating how effective management mitigates risks. This exposure disparity is driven by intersecting factors: high population density in developing regions amplifies pollution, while climate extremes exacerbate scarcity in arid zones [9].

The GlakeWQ framework (available at https://github.com/ShenMing93/RS2WQ) enables scientists and local governments to transform these findings into actionable policy tools. By providing open-access, high-resolution (100 m) water quality maps, the framework enables three key applications:

**Cost efficiency: revolutionizing monitoring economics.** Traditional SDG 6.3.2 assessments require substantial investment for *in situ* measurement of five physicochemical parameters, including the cost of equipment, trained personnel, and technical expertise—expenses often unaffordable for low- and middle-income countries. For instance, in Malawi (GDP about USD 12 billion), the 2023/24 water budget accounts for less than 0.3% of GDP, leaving the country heavily dependent on external aid and severely limiting its capacity for routine water quality monitoring. As a result, fewer than 40% of UN member states can provide comprehensive SDG 6.3.2 data. GlakeWQ provides a globally scalable, cost-effective alternative by leveraging automated Sentinel-2 satellite processing. Compared with traditional *in situ* methods, GlakeWQ significantly reduces costs related to equipment, personnel, and technical expertise, thus enabling low- and middle-income countries to perform frequent water quality assessments. The savings can be reinvested in water protection, management, and governance, improving both the efficiency of water management and overall water quality.

**SDG reporting reform: closing the 70-nation data gap.** Building on this cost-effective foundation, the GlakeWQ framework can seamlessly integrate with UN-Water’s SDG 6 Data Portal by creating the first standardized global lake water quality database for 70 previously unreported nations. Most of these are low-income countries in Africa (e.g. Tunisia, Djibouti) and Asia (e.g. Cambodia, Bangladesh). This addresses the major monitoring gap highlighted in the 2024 UN SDG report, where these regions previously submitted no data for indicator 6.3.2 on lake water quality. Our framework delivers globally consistent, quarterly 100-m resolution water quality maps for 16 409 lakes, which directly feed into UN reporting systems. Crucially, it resolves the long-standing ‘optical signature gap’ for nonoptically active parameters such as DO, through machine learning linkages validated at 75.4% accuracy against China’s GB3838-2002 standard (Fig. S4). This capability enables SDG scoring for previously unmonitored lakes such as Tonlé Sap (Cambodia) and Lake Saumatre (Haiti), as well as transboundary lakes like Lake Turkana (Kenya/Ethiopia) and Lake Peipsi (Russia/Estonia), where joint governance agencies are lacking or defunct. It thus transforms policy paralysis into actionable water governance.

**High-impact interventions: targeted resource allocation enabled by GlakeWQ.** Beyond monitoring and reporting, the GlakeWQ framework supports targeted interventions by prioritizing regions where severe water degradation overlaps with high population exposure. A key strength lies in its ability to distinguish between lakes that require costly restoration and those still suitable for protection, which is central to cost-effective management. For instance, despite ongoing restoration efforts since 1972, Lake Erie continues to experience harmful algal blooms under the combined pressures of climate change and human activities (Fig. 1c and d). Likewise, Lake Taihu experienced rapid deterioration during China’s post-reform economic expansion. Although substantial investments in recent years have partially improved water quality, the lake remains highly eutrophic with recurrent blooms (Fig. 1c and d). These examples highlight that restoration is lengthy, costly, and uncertain, whereas proactive protection of relatively healthy systems is far more efficient [10]. Lake Victoria illustrates this urgency (Fig. 1c and d), as its overall water quality remains relatively good, yet unregulated development and pollution over the past two decades have triggered early-stage degradation, with some bays already experiencing frequent algal blooms. By identifying such tipping points, GlakeWQ empowers policy-makers and resource managers to implement timely interventions that safeguard water quality before irreversible damage occurs.

Our findings reveal that global freshwater systems reflect a fundamental dimension of planetary inequality. By integrating multi-source satellite observations, environmental standards, and machine learning, GlakeWQ transforms fragmented local monitoring into a unified global framework—making freshwater quality a measurable and comparable indicator of development equity. The identification of dual degradation pathways, namely, climate forcing (e.g. temperature and precipitation changes) and human activities (e.g. urbanization, industrial discharge, and land-use changes), highlights their intertwined roles in shaping water security, providing an empirical foundation for global hydrological justice.

Importantly, our results demonstrate that restoration, while necessary, remains costly, slow, and uncertain, whereas proactive protection of relatively healthy systems yields far greater long-term benefits. This insight supports a paradigm shift in freshwater governance—from reactive remediation toward anticipatory protection guided by near-real-time Earth observation.

Looking forward, extending GlakeWQ to include real-time climate risk modeling (e.g. ENSO- and extreme-weather–driven bloom prediction) and small-lake monitoring for rural communities will further bridge the gap between scientific discovery and equitable policy action. In doing so, freshwater management can move from localized projects to planetary governance, where environmental sustainability and social justice converge.

## Supplementary Material

nwag445_Supplemental_File
