# Supplementary material for: Satellite mapping exposes global inequities in lake water quality across 145 countries
Source: Natl Sci Rev. 2026 Jul 20;13(15):nwag445. doi: 10.1093/nsr/nwag445 (PMC13471035; doi:10.1093/nsr/nwag445)
Supplement: Supplementary file 1 [file nwag445_supplemental_file.docx]

**Supplementary materials for “Satellite Mapping Exposes Global Inequities in Lake Water Quality Across 145 Countries”**

Ming Shen ^a, b^, Tianci Qi ^a^, Juhua Luo ^a, b, c^, Zhigang Cao ^a^, Wei Zhi ^d^, Yunlin Zhang ^a, b, c^, Daniel A. Maciel ^e^, Tiit Kutser ^f^, Anthony Gidudu ^g^, Zhe Sun ^a, b, c^, Steven Loiselle ^h^, Hongtao Duan ^a, b, c *^

^a^ State Key Laboratory of Lake and Watershed Science for Water Security, Nanjing Institute of Geography and Limnology, Chinese Academy of Sciences, Nanjing 211135, China

^b^ University of Chinese Academy of Sciences, Nanjing 211135, China

c University of Chinese Academy of Sciences, Beijing 100049, China

^d^ The National Key Laboratory of Water Disaster Prevention, Yangtze Institute for Conservation and Development, Key Laboratory of Hydrologic-Cycle and Hydrodynamic-System of Ministry of Water Resources, Hohai University, Nanjing 211100, China

^e^ Instrumentation Lab for Aquatic Systems (LabISA), Earth Observation Coordination of National Institute for Space Research (INPE), São José dos Campos/SP, 12227-010, Brazil

^f^ Estonian Marine Institute, University of Tartu, Mäealuse 14, 12618 Tallinn, Estonia.

^g^ Department of Geomatics and Land Management, Makerere University, P.O. Box 7062, Kampala, Uganda

^h^ Italy Dipartimento Farmaco Chimico Tecnologico, CSGI, University of Siena, 53100 Siena, Italy

* Corresponding author: Hongtao Duan. Email: [htduan@niglas.ac.cn](mailto:htduan@niglas.ac.cn)

**Contents of this file**

1. Text S1 to S5

2. Figures S1 to S10

3. Tables S1 to S4

# Text S1 Data sources

***Sentinel-2 MSI data***. The MultiSpectral Instrument (MSI) onboard the Sentinel-2 satellites (S2A and S2B) offers global coverage within a 5-day revisit frequency and provides valuable data for monitoring inland water quality. The S2 MSI surface reflectance (SR) products [1] are computed with a Sen2Cor processor algorithm for atmospheric correction and a scene classification (SCL) algorithm for generating a classification map. Additionally, the S2 cloud probability products [2] are created using a LightGBM algorithm to identify and classify clouds and highly reflective surfaces, which assign a probability value from 0 to 100, with higher values indicating a higher likelihood of being clouds or reflective surfaces. Both S2 SR and cloud probability products, initially provided by the European Space Agency (ESA) Copernicus Programme, are all available in Google Earth Engine. The archive of S2-MSI Level-2A SR products [1] since 2019 (when L2 coverage is available globally) covering studied lakes was used.

***Lake shapefile.*** We used lake boundaries provided by HydroLAKES [3] to determine the lakes examined in our study. Lakes with an area greater than 10 km^2^ were selected in our study. Moreover, lakes without valid imagery were excluded after quality control. A total of 16,409 lakes (≥10 km²) across 145 countries were analyzed (Figure S3). The number of lakes per country averages 113±631, ranging from a single lake in several nations (e.g., Tonga, Djibouti, and Saudi Arabia) to a maximum of 7,159 lakes in Canada.

***In situ data.*** We gathered *in situ* measurements from various sources to train and validate the water quality assessment model (Figure S4g), including field surveys, data sharing, and publicly available datasets. These data encompassed a vast range of inland and coastal optical regimes, with chlorophyll-a (Chl*a*) concentrations spanning four orders of magnitude (0.058 to 660.7 μg L^-1^) and Secchi disk depth (SDD) ranging from 0.01 m in extreme turbid waters to 45 m in ultra-clear waters.

These datasets can be mainly divided into two categories. The first category was the optically active water quality parameters dataset [4-9], i.e., Chl*a*, suspended particulate matter (SPM), the absorption coefficient of colored dissolved organic matter (CDOM) at 440 nm (*a*_g_440), and SDD. After the concurrent match with a time window of ±12 h, the number of field-satellite matchups for Chl*a*, SPM, CDOM, and SDD was 966, 757, 569, and 832, respectively.

The second category was the dataset of water quality class. The water quality classes are determined based on five physicochemical water quality parameters according to China’s surface water quality standards (GB3838-2002) (see Definition of good water quality and statistical analysis below). These data were obtained from the Ministry of Ecology and Environment in China ([https://www.mee.gov.cn](https://www.mee.gov.cn/)), the United States National Lakes Assessment (NLA) (<https://www.epa.gov>), the National Network for the Monitoring of the Waters of the Lakes of Greece (<http://biodiversity-info.gr/index.php>), the Brazilian National Water Agency ([https://www.snirh.gov.br](https://www.snirh.gov.br/)), the Waterbase of European Environment Agency (<https://www.eea.europa.eu>), and the Global Freshwater Quality Database (GEMStat) (<https://gemstat.org>). It should be noted that many of the data do not include all five needed physicochemical parameters, and their water quality classes were determined based on the available physicochemical parameters. After the concurrent match with a time window of ±12 h, the number of field-satellite matchups for water quality classes was 10,957.

***UN report data.*** The UN reported SDG 6.3.2 scores obtained from the SDG 6 Data Portal (<https://sdg6data.org>) were also used for comparison at the country scale. Among the 145 countries included in our study, a total of 75 countries provided reporting values for lake water quality.

***Auxiliary data.*** The list of developed countries was obtained from the *World Economic Outlook* by the International Monetary Fund [10]*,* and the list of least-developed countries was obtained from the UN Trade and Development [11]; all remaining countries were classified as developing countries. Long-term water quality data for Lake Erie were obtained from the Great Lakes Water Quality Monitoring and Surveillance Data (<https://data-donnees.az.ec.gc.ca/>), and for Lake Taihu from the Lake Taihu Laboratory Ecosystem Research (TLLER) (<http://thl.cern.ac.cn/>). Algal bloom frequency for Lake Victoria was obtained from [12]. GDP per capita data were obtained from the World Bank (<https://data.worldbank.org/>). The global population data was obtained from the WorldPop program [13, 14].

# Text S2 Preprocessing of S2 MSI data

***Upscaling and downloading***. We aggregated the MSI SR data to a spatial resolution of 100 meters on the GEE platform. Upscaling helps enhance the signal-to-noise ratio (SNR) to meet the minimum requirements for water color [15, 16]. Additionally, it could significantly improve computational efficiency. This resolution of 100 meters fulfills the user requirements for climate and general applications [17].

***Waterbody*** ***identification and mask.*** The sub-images of each lake were obtained using the bounding rectangle of the fixed lake shapefiles. The water pixels were identified using the SCL band in the MSI SR products. The cloud and highly reflective pixels were removed using the S2 cloud probability band with a probability of larger than 20%. The surface algal scums and aquatic vegetation pixels were further excluded using the floating algae index (FAI) with a threshold of 0.005 [18]. The water boundaries thus identified were eroded by two aggregated pixels (~200 m) buffer into the lake interior to mitigate the potential mixed land-water pixels and land adjacency effects (LAE) [19].

***Remote sensing reflectance correction***. MSI SR products were atmospherically corrected by Sen2Cor, which has been successfully applied in inland water color studies [20, 21]. However, Sen2Cor was initially developed for land applications, which does not consider the unique sky- (or sun-) glint effect, leading to an inevitable overestimation over water, especially for clean waters [20]. Pure water exhibits strong absorption in the near-infrared (NIR) or short-wave infrared (SWIR) bands, and water-leaving reflectance should be negligible at the NIR (for clear water) or SWIR (for turbid water) bands [22]. Therefore, we can assume that the NIR and SWIR signals are attributed to the sky- (or sun-) glint and other noise (e.g., residual aerosol scattering). A simple method of subtracting the minimum reflectance at the NIR and SWIR bands is adopted to remove the residual noise in the water-leaving signals [21, 23]. The noise-corrected reflectance is then converted to remote sensing reflectance (*R_rs_*) by dividing by π. The correction formula is as follows:

$R_{rs}\left( \lambda\right)=\frac{R\left( \lambda\right)-min(R\left( 865 \right), R\left( 1610 \right), R(2202))}{\pi}$ , (1)

where *R_rs_*(λ) is the remote sensing reflectance at λ nm, *R*(λ) is the Sen2Cor-derived surface reflectance at λ nm, and min(*R*(865), *R*(1610), *R*(2202)) is the minimum surface reflectance at the NIR (865 nm) and SWIR (1610 nm and 2202 nm) bands. After the abovementioned correction, the MSI-derived *R_rs_* achieved good performances in comparison with *in situ* measured *R_rs_* from the first category dataset, with *R*^2^ ranging from 0.50 to 0.89 and the MAPE ranging from 22.9% to 61.4% (Figure S9).

# Text S3 Definition and statistical analysis of good water quality

Water quality classes were determined according to China’s surface water quality standards (GB3838-2002) [24], using five physicochemical water quality parameters (i.e., pH, TP, DO, COD, and NH_4_^+^-N). These parameters can reflect the main water quality impairments present in many regions worldwide and are also fundamental parameters in water quality assessment frameworks of the United Nations [25], United States of America (USA) [26], the European Union [27], and others [28, 29] (Tables S1-2). Each physicochemical indicator is classified into six levels (Table S1). The highest level of the physicochemical indicator (indicating the most polluted) at a specific monitoring site sets the overall water quality level. These six levels can be aggregated into two classes, namely, good classes (levels I to III) and poor classes (levels IV to inferior to V). According to GB3838-2002, the water quality of centralized drinking water sources (CDWS) must fall into the good classes, ensuring they are suitable for consumption after conventional treatment.

The spatial and temporal statistics for water quality classes were conducted as follows (Figures S1-S2) [25, 30]. (1) Pixel scale: if the proportion of time when water is of good quality (i.e., compliance duration/total duration) is greater than 80%, the water quality of this pixel during the monitoring period is “good”. (2) Lake scale: first, the water quality class of each pixel in the lake during the monitoring period was determined as mentioned above; then, if the proportion of area with good water quality in the lake (i.e., number of good quality pixels/total number of pixels) is greater than 80%, then the water quality in the lake during the monitoring period is “good”. (3) National scale: first, the water quality status of the pixel scale and the lake scale was determined as mentioned above; then, the proportion of lakes with good water quality was calculated for the country as the national score of indicator SDG 6.3.2. Here, the scores above 80% indicated a high level of progress towards the SDG 6.3.2 target, scores between 50% and 80% indicated a medium level, and scores below 50% indicated a low level

# Text S4 Two-stage random forest water quality assessment model

***Model development****.* With over ten thousand *in situ* measurements, a two-stage random forest water quality assessment model was developed to bridge the optical satellite observations and the water quality management (Figure S1). Random forest (RF) is an ensemble learning method that constructs multiple decision trees and combines their results for prediction or classification [31]. The random forest method is widely used in remote sensing of inland water bodies [21, 30] due to its robustness against random noise and outliers achieved through random sampling and feature selection [31].

The assessment model consists of two stages (Figure S1). i) In the first stage, the random forest regression method (RFR-OWQPs) was used to determine the four OWQPs (i.e., Chl*a*, SPM, *a_g_*440, and SDD) from satellite-derived *R_rs_*. Input variables include nine bands of MSI from visible to near-infrared (NIR) (443, 492, 560, 665, 704, and 740 nm) and five band ratios commonly used for water quality estimation, including 443/560, 492/560, 665/560, 704/560, and 704/665. ii) In the second stage, the random forest classification method (RFC-WQC) was used to explore the nonlinear responses between physicochemical parameters-determined water quality classes (i.e., good or poor) and OWQPs obtained in the first stage. A K-fold (K=5 here) method was adopted for cross validation. The hyperparameters (i.e., n_estimators, max_features, max_depth) in each fold are optimized by the Bayesian method [32]. In this study, the model was implemented using the Python scikit-learn package.

***Model validation and comparsion****.* The two-stage model demonstrated high accuracy and reliability, as supported by extensive validation results (Figure S4). In the first stage (Rrs2OWQPs), the 5-fold validation exhibited high accuracy in the four optical water quality parameter models (Figures S4a-d). The Chl*a* inversion yielded an R^2^ of 0.79, RMSE of 20.32 μg L^-1^, MAPE of 27.5% (N=966). The SPM inversion achieved an R^2^ of 0.73, RMSE of 13.28 mg L^-1^, and MAPE of 26.1% (N=757). The *a_g_*440 inversion resulted in an R^2^ of 0.66, RMSE of 0.96 m^-1^, and MAPE of 31.3% (N=569). Lastly, for SDD, an R^2^ of 0.81, RMSE of 0.79 m, and MAPE of 12.6% (N=832) were obtained.

We also compared the RFR-OWQPs model with existing state-of-the-art empirical, semi-analytical, and machine learning algorithms. Chla: GU2B [33], OC3 [34], Blend [35], and Gons [36]; SPM: Miller [37], Xue [38], Hou [19], GAA [39], Nechad665 [40], and Nechad704 [40]; *a*_g_440: Kutser [41], Mannino [42], Liu [20], and QAA-CDOM [43]; SDD: Shi [44], Caballero [45], Ren [46], Alikas [47], and Lee [48]. The machine learning algorithms included support vector regression [49] and extreme gradient boosting (XGB) [50] for all OWQPs. The results revealed the superior performance of the RFR-based OWQP model with higher R^2^ and Lower RMSE and MAPE, compared to commonly used empirical, semi-analytical, and other machine learning algorithms (Figure S5).

In the second stage (OWQPs2WQC), the 5-fold validation demonstrated satisfactory classification performance (overall accuracy (OA) of 75.4%, N=10,957) (Figure S4e). All the machine learning-based classification models performed well (OA>73.5%), with the RFC-based model exhibiting the highest performance (Table S3). Notably, according to the importance analysis of the random forest classification model, SDD emerged as the most influential OWQP for determining water quality classes based on optical measurements, followed by Chl*a*, SPM, and *a_g_*440 (Figure S4f).

We evaluated model performance across different OWQPs (Figure S6). Overall, the model maintained satisfactory accuracy (OA ≥ 65%). The model exhibited a characteristic U-shaped pattern of performance for Chl*a*, SPM, and SDD, with higher accuracy at both low and high values (OA ranging from 70.6% to 91.2%) and reduced accuracy in intermediate ranges (OA ranging from 68.1% to 72.2%). Low values generally correspond to “good” water quality in clear, oligotrophic-mesotrophic lakes (Chl*a* ≤ 10 μg L^-1^, SPM ≤ 10 mg L^-1^, SDD ≥ 2 m), while high values indicate “poor” water quality in eutrophic or turbid lakes (Chl*a* ≥ 50 μg L^-1^, SPM ≥ 50 mg L^-1^, SDD ≤ 1 m). Intermediate ranges (moderate eutrophic or slightly turbid waters) showed somewhat lower accuracy. For CDOM, the model showed reduced accuracy at high values, likely due to strong light absorption, which produces dark signals that resemble clear water. We also tested the model on lakes across different continents and found that the spatial distribution of water quality aligned well with the observed patterns in these bodies of water (Figure S7). Therefore, the model performed robustly across most lake types, achieving satisfactory accuracy for global-scale mapping.

The mean absolute difference between the SDG 6.3.2 scores obtained through MSI observations and the latest UN reported values was 43.4% (Figure S8). These differences may mainly arise from variations in assessment criteria, monitoring periods, the number of monitored water bodies, and data quantity and quality. Generally, rich countries with extensive monitoring programs could submit data covering more water bodies with higher data quality, with 40 of the highest-income countries contributing to 75% of reported water bodies, while only 1% of data were in the poorest 20 countries [51]. Therefore, the differences were lower in developed countries (30.3%) than in developing countries (41.8%) or least-developed countries (66.6%) (Figure S8). In most developed countries, differences lower than 30% were observed, particularly in countries with robust monitoring programs where differences were below 10% (Figure S8).

***Model Advantages***. We have introduced a two-stage water quality assessment model with machine learning techniques. This approach established a correlation between optical remote sensing observations and physicochemical parameters-determined water quality classes. Compared to traditional *in situ* measurement methods, our model offers a cost-effective and efficient means to glean insights into global lake water quality. Our extensive database transcends temporal and spatial constraints, encompassing 145 countries worldwide. Although currently limited to medium and large-sized lakes (>10 km²) due to satellite limitations, these data serve as invaluable scientific benchmarks to assist governments and stakeholders engaged in lake management. This is especially beneficial for regions grappling with constrained water-related budgets or deficient monitoring infrastructure [52, 53].

Given the inherent variability of water bodies and the unique conditions prevailing in different nations, it is crucial to recognize that the choice of water quality parameters and their respective target values may vary [28], tailoring them to the specific needs of each country or water body. However, disparities may emerge when evaluating shared transboundary water bodies if neighboring nations adhere to divergent target values [28, 54]. For instance, there are notable variations in water quality monitoring and assessment methods between the sharing countries (Estonia and Russia) for the transboundary Lake Peipsi [55, 56]; therefore, a Joint Committee was established to guarantee measurement consistency. Nevertheless, ongoing regional conflicts/wars impeded the commission’s efforts and hindered consistent data acquisition. Additionally, the reported values for different countries within the same data archive may originate from diverse monitoring periods. Even within the same country, reports spanning the data archives might pertain to varied monitored water bodies [51, 57]. Moreover, countries might selectively present their data to secure international funding or to project a more favorable national image for political purposes. Consequently, these influences have considerably impeded the objectivity and comparability of the data reported by the UN.

In contrast, our database provides unbiased and globally comparable insights into lake water quality by adhering to a uniform methodology and water quality assessment criteria (leveraging China’s standards here). As a result, stakeholders can attain a coherent comprehension of lake water quality and make well-informed decisions to safeguard lakes with good water quality and improve those grappling with poor conditions. Furthermore, it is possible to customize our assessment model to align with alternative local or international standards or incorporate additional water quality parameters as required.

***Model uncertainties***. It is essential to recognize that the assessment model was formulated by exploring both the potential and weak linkages between optically active parameters and optically non-active physicochemical parameters (Figure S10). In cases where the response exceeds weak correlations or when optically active parameters fail to reflect alterations in physicochemical parameters, it could lead to inaccuracies in water quality classification, impeding the utility of our model. There was also a discrepancy in model performance for “good” (recall=54.9%) and “poor” (recall=88.4%) water quality classes (Figure S4e and Table S3). The discrepancy likely stems from the classification logic of China’s surface water quality standards (GB3838-2002) and the inherent limitations of remote sensing for non-optical parameters. In good water bodies, all five physicochemical parameters are consistently favorable, yielding a relatively homogeneous signal that the model can effectively identify. In contrast, in poor water bodies, water quality may be driven by a single degraded physicochemical parameter, while others remain good, resulting in highly heterogeneous and complex optical signatures. Given that correlations between individual physicochemical parameters and OWQPs were generally weak, isolated fluctuations in a single non-optical parameter may not be sufficiently reflected in OWQPs, leading to higher misclassification rates and limiting accuracy for poor water quality.

Another potential source of uncertainty in the model arose from the propagation of OWQPs estimation errors into the water quality classification task. To quantify these uncertainties, we conducted a Monte Carlo simulation in which stochastic perturbations were drawn for each sample from a normal distribution with a standard deviation equal to the MAPE of the corresponding input OWQP. The simulation results indicated that across all tested input variable combinations, the OA of classification remained stable at 73.8%-75.0% (Table S4). Even when uncertainties in all four OWQPs were considered simultaneously, the OA remained as high as 73.76%, nearly identical to the original model’s performance (75.4%). These findings demonstrated that, despite estimation errors in OWQPs, the binary classification of “Good” versus “Poor” water quality was robust. This stability arose partly because the model was trained on a large number of global in situ observations, providing strong generalization ability, and partly because minor numerical fluctuations in OWQPs typically remain within the same broad water quality category.

Moreover, due to limitations in satellite observations and computational constraints, the lack of monitoring for small lakes (<10 km^2^) has, to some extent, restricted the comprehensiveness of our data and is also a significant factor contributing to the discrepancies between our results and those in the UN reports. This is partly because the UN reporting framework relies on whatever in situ data each country provides and does not specify a minimum lake size, so small lakes may be included in some regions but absent in others. While our current focus ensured high data integrity for the lakes (≥10 km2) that constitute ~75% of global lake area [3], we acknowledge that smaller lakes are vital components of local ecosystems. In the future, expanding coverage to include smaller lakes and developing more robust water quality assessment models that integrate watershed characteristics, climatic conditions, and other relevant data could prove instrumental in effectively tackling these challenges [58].

# Text S5 Data Availability Statement

The Sentinel-2 MSI data were avalible in google earth engine (<https://earthengine.google.com/>). The main results produced by this study are available from <https://doi.org/10.57760/sciencedb.40742>. The source code used in this study was available at the repository (<https://github.com/ShenMing93/RS2WQ>).

**Fig. S1. Schematic flow for compilation of the global lake water quality (GlakeWQ) database employing the two-stage random forest water quality model.** The two-stage random forest water quality assessment model was developed with over ten thousand *in situ* measurements and can be divided into two stages. i) In the first stage, we established inversion algorithms using the random forest regression method (RFR-OWQPs) from satellite-derived *R_rs_* for optically active water quality parameters, i.e., Chl*a*, SPM, *a_g_*440, and SDD. ii) In the second stage, the random forest classification method (RFC-WQC) was used to establish the nonlinear response relationship between physicochemical parameters-determined water quality classes (i.e., good or poor) and OWQPs obtained in the first stage.

**Fig. S2. Statistics analysis of lake water quality.** A two-stage random forest water quality model was employed to compile the global lake water quality (GlakeWQ) database. According to China’s surface water quality standards (GB3838-2002), water quality classes for each station were determined using five physicochemical water quality parameters (i.e., pH, TP, DO, COD, and NH_4_^+^-N) (see Supplementary Table 1), with water quality levels I-III considered good, while levels IV-inferior V regarded as poor. A lake with good water quality means most of its water areas can maintain long-term stable good water quality. Therefore, the spatial and temporal statistics for water quality classes were conducted as following steps [25, 30]. (1) Pixel scale: if the proportion of time when water is of good quality (i.e., compliance duration/total duration) is greater than 80%, the water quality of this pixel during the monitoring period is “good”. (2) Lake scale: first, the water quality class of each pixel in the lake during the monitoring period was determined as mentioned above; then, if the proportion of area with good water quality in the lake (i.e., number of good quality pixels/total number of pixels) is greater than 80%, then the water quality in the lake during the monitoring period is “good”. (3) Country scale: first, the water quality status of the pixel scale and the lake scale was determined as mentioned above; then, the proportion of lakes with good water quality was calculated for the country as the national score of indicator SDG 6.3.2.

**Fig. S3. Geographic distribution of lakes (>10 km^2^) and the total number of lakes per country.**

**Fig. S4. Validation of the two-stage random forest water quality assessment model using *in situ* measurements. a-d**, Performance of random forest regression model for OWQPs (RFR-OWQPs), including RFR-Chl*a*, RFR-SPM, RFR-*a_g_*440, and RFR-SDD models. **e**, Confusion matrix and metric of RFC-WQC model. **f**, Relative feature importance of RFC-WQC model. **g**, Locations of the *in situ* measurements. Orange circles denote optically active water quality parameters (OWQPs) measurements, and green circles refer to the water quality classes (WQC) measurements.

**Fig. S5. Comparison with the performance of random forest regression model for OWQPs (RFR****-OWQPs) and state-of-art OWQP algorithms. a**, Chl*a*. **b**, SPM. **c**, *a_g_*440. **d**, SDD. The units of RMSE for Chl*a*, SPM, *a_g_*440, and SDD are μg L^-1^, mg L^-1^, m^-1^, and m, respectively. SVR: Support Vector Regression; XGB: eXtreme Gradient Boosting; RFR: Random Forest Regression.

**Fig. S6. Performance of the RFC-WQC model under different OWQPs. a,** Chl*a*; **b**, SPM; **c**, *a_g_*440; and **d**, SDD.

**Fig. S7. Examples showing the spatial pattern for the proportion of time (%) maintaining good water quality in typical lakes across different continents.** **a**, Great Lakes in the USA and Canada, **b**, Lake Peisi in Russia and Ukraine, **c**, Lake Balaton, in Hungary, **d**, Lake Bay in the Philippines, **e**, African Great Lakes in Congo-Kinshasa, Uganda, Kenya, Ethiopia, Rwanda, Burundi, Tanzania, Zambia, Malawi, and Mozambique, **f**, Lake Taihu and surrounding small lakes in China, **g**, Dense lakes in Nunavut, Canada, **h**, Lake Summer and Lake Abert in the Great Basin in the USA, **i**, Dense lakes in Qiangtang terrane, China, and **j**, Lake Disappointment and surrounding small lakes in the Gibson Desert in Australia. The base map was the population obtained from the WorldPop program [13, 14].

**Fig. S8. Comparison of SDG 6.3.2 scores between our database and the UN reported values in 2023 or the latest available year.** The boxplots were statistics of absolute differences in different development stages of countries. The scatter plot was the SDG 6.3.2 scores between our database and the UN reported values in developed countries. The SDG 6.3.2 score of each country was defined as the proportion of lakes with good water quality. The UN reported values were obtained from the SDG 6 Data Portal (https://sdg6data.org), with 75 countries (out of 145 studied countries) providing officially reported values for lake water quality.

# Fig. S9. Validation of MSI-derived *R_rs_* from the SEN2COR algorithm and a simple residual noise corrections scheme (N=718). a, *R_rs_*(443), b, *R_rs_*(492), c, *R_rs_*(560), d, *R_rs_*(665), e, *R_rs_*(704), and f, *R_rs_*(740)

**Fig. S10.** Differences of OWQPs in physicochemical parameters-determined water quality classes (WQC). **a**, Chla; **b**, SPM; **c**, ag(440); **d**, SDD.

**Table S1. Water quality standards for surface waters in China (GB3838-2002)**. These six levels can be aggregated into two classes, namely, good classes (levels I to III) and poor classes (levels IV to inferior to V).

| Class | Good | | | Poor | | |
| --- | --- | --- | --- | --- | --- | --- |
|  | I | II | III | IV | V | Inferior to V |
| pH (dimensionless) | [6.0, 9.0] | | | | | <6 or >9 |
| TP (mg L^-1^) | ≤0.01 | (0.01,0.025] | (0.025,0.05] | (0.05,0.1] | (0.1, 0.2] | >0.2 |
| NH_4_^+^-N (mg L^-1^) | ≤0.15 | (0.15, 0.5] | (0.5, 1.0] | (1.0, 1.5] | (1.5, 2.0] | >2.0 |
| DO (mg L^-1^) | ≥7.5 | [6.0, 7.5) | [5.0, 6.0) | [3.0, 5.0) | [2.0, 3.0) | <2.0 |
| COD (mg L^-1^) | ≤2.0 | (2.0, 4.0] | (4.0, 6.0] | (6.0,10.0] | (10.0,15.0] | >15.0 |

**Table S2. Comparison of surface water quality standards for different countries ^1^.**

| Country/Region | pH (dimensionless) | TP (mg L^-1^) | NH_4_^+^-N (mg L^-1^) | DO (mg L^-1^) | COD (mg L^-1^) |
| --- | --- | --- | --- | --- | --- |
| China | [6.0, 9.0] | ≤0.05 | ≤1.0 | ≥5.0 | ≤6.0 |
| England/Wales | [6.5, 9.5] | ≤0.049 | ≤0.6 | ≥6.0 | - |
| Germany | [6.5, 9.5] | ≤0.15^2^ | ≤0.3 | ≥6.0 | TOC^3^≤5.0 |
| US^4^ | [5.0, 9.0] | ≤0.027^5^ | ≤1.9^6^ | ≥5.5 | - |
| Australia | [6.5, 9.0] | ≤0.05 | ≤1.49^6^ | ≥6.0 | - |
| Russia | [6.5, 8.5] | ≤0.2^1^ | ≤0.5 | ≥6.0 | ≤4.0 |
| Japan | [6.5, 8.5] | ≤0.01 | - | ≥5.0 | ≤3.0 |
| South Korea | [6.5, 8.5] | ≤0.03 | - | ≥5.0 | ≤4.0 |
| Vietnam | [6.5, 8.5] | ≤0.3 | ≤0.3 | ≥6.0 | ≤4.0 |
| India | [6.0, 8.5] | - | - | ≥4.0 | BOD^7^≤3.0 |
| Philippines | [6.0, 9.0] | ≤0.5^2^ | ≤0.05 | ≥5.0 | BOD≤3.0 |
| Thailand | [5.0, 9.0] | - | ≤0.5 | ≥4.0 | BOD≤2.0 |
| Uganda | [5.5, 8.5] | - | ≤0.5 | - | - |
| Kenya | [6.5, 8.5] | - | ≤0.5 | - | - |
| Brazil | [6.0, 9.0] | ≤0.05 | ≤0.5 | ≥5.0 | BOD≤5.0 |

^1^ It should be noted that for countries with multi-tiered water quality classifications, the threshold was selected based on the specific category defined as suitable for use as the drinking water supply.

^2^ Rivers and lakes were not distinguished.

^3^ Refers to Total Organic Carbon.

^4^ Refers to the National Recommended Water Quality Criteria on Aquatic Life of USEPA.

^5^ Dynamic threshold; the value presented here is an illustrative example derived from the Huron/Erie Lake Plains ecoregion, conditioned on a target Chl*a* concentration of 10 μg L^-1^ at a 95% certainty level.

^6^ Dynamic threshold; the value was at pH=7 and temperature=20℃.

^7^ Refers to Biochemical Oxygen Demand.

**Table S3. Comparison of different machine learning algorithms for water quality classification^*^.**

|  | Class | Poor | Good | Precision (%) | OA (%) |
| --- | --- | --- | --- | --- | --- |
| **DT-WQC** | Poor | 2147 | 723 | 74.8 | 74.3 |
|  | Good | 2092 | 5995 | 74.1 |  |
|  | Recall (%) | 50.6 | 89.2 |  |  |
| **KNN-WQC** | Poor | 2228 | 789 | 73.85 | 74.4 |
|  | Good | 2011 | 5929 | 74.67 |  |
|  | Recall (%) | 52.6 | 88.3 |  |  |
| **LR-WQC** | Poor | 2277 | 798 | 74.1 | 74.8 |
|  | Good | 1962 | 5920 | 75.1 |  |
|  | Recall (%) | 53.7 | 88.1 |  |  |
| **NB-WQC** | Poor | 2146 | 671 | 76.18 | 74.8 |
|  | Good | 2093 | 6047 | 74.28 |  |
|  | Recall (%) | 50.6 | 90.0 |  |  |
| **DNN-WQC** | Poor | 2015 | 660 | 75.32 | 73.7 |
|  | Good | 2224 | 6058 | 73.2 |  |
|  | Recall (%) | 47.5 | 90.2 |  |  |
| **XGB-WQC** | Poor | 2316 | 814 | 74.0 | 75.0 |
|  | Good | 1923 | 5904 | 75.4 |  |
|  | Recall (%) | 54.6 | 87.9 |  |  |
| **SVM-WQC** | Poor | 2082 | 647 | 76.29 | 74.4 |
|  | Good | 2157 | 6071 | 73.78 |  |
|  | Recall (%) | 49.1 | 90.4 |  |  |
| **RFC-WQC** | Poor | 2327 | 779 | 74.9 | **75.4** |
|  | Good | 1912 | 5939 | 75.7 |  |
|  | Recall (%) | 54.9 | 88.4 |  |  |

^*^DT: Decision Tree; KNN: K-Nearest Neighbor; LR: Logistic Regression; NB: Naive Bayes; DNN: Deep Neural Network; XGB: eXtreme Gradient Boosting; SVM: Support Vector Machine; RFC: Random Forest Classification

**Table S4. Model sensitivity of second stage (OWQPs2WQC) to OWQPs estimation uncertainties at the first stage (Rrs2OWQPs) ^1^.**

| Variables with uncertainty | Precision (%) | | Recall (%) | | OA (%) |
| --- | --- | --- | --- | --- | --- |
|  | Good | Poor | Good | Poor |  |
| Chl*a* | 75.2 | 73.8 | 87.9 | 54.2 | 74.8 |
| SPM | 75.2 | 74.2 | 88.1 | 54.0 | 74.9 |
| *a_g_*(440) | 75.3 | 74.2 | 88.1 | 54.1 | 75.0 |
| SDD | 75.3 | 74.0 | 88.0 | 54.3 | 75.0 |
| Chl*a*, SPM | 75.0 | 73.2 | 87.6 | 53.7 | 74.5 |
| Chl*a*, *a_g_*(440) | 75.0 | 73.1 | 87.6 | 53.7 | 74.4 |
| Chl*a*, SDD | 75.1 | 73.1 | 87.5 | 54.1 | 74.6 |
| SPM, *a_g_*(440) | 75.0 | 73.6 | 87.9 | 53.4 | 74.6 |
| SPM, SDD | 75.0 | 73.4 | 87.8 | 53.5 | 74.5 |
| *a_g_*(440), SDD | 75.1 | 73.6 | 87.8 | 54.0 | 74.7 |
| Chl*a*, SPM, *a_g_*(440) | 74.8 | 72.8 | 87.4 | 53.2 | 74.2 |
| Chl*a*, SPM, SDD | 74.8 | 72.5 | 87.2 | 53.4 | 74.1 |
| Chl*a*, *a_g_*(440), SDD | 74.8 | 72.6 | 87.2 | 53.5 | 74.2 |
| SPM, *a_g_*(440), SDD | 74.8 | 72.9 | 87.5 | 53.4 | 74.3 |
| Chl*a*, SPM, *a_g_*(440) , SDD | 74.5 | 71.9 | 86.9 | 52.9 | 73.8 |

^1^ A Monte Carlo analysis was conducted to quantify how Stage 1 OWQPs uncertainties influence Stage 2 water quality classification. For each sample, stochastic perturbations were drawn from a normal distribution with a standard deviation equal to the MAPE of the corresponding input variable. The Monte Carlo analysis was performed over 1000 simulations.

# References

1.	Main-Knorn M, Pflug B, Louis J *et al.* Sen2Cor for Sentinel-2. In: *ProcSPIE,* *2017*.

2.	Zupanc A. *Improving cloud detection with machine learning*. <https://medium.com/sentinel-hub/improving-cloud-detection-with-machine-learning-c09dc5d7cf132024>, date last accessed).

3.	Messager ML, Lehner B, Grill G *et al.* Estimating the volume and age of water stored in global lakes using a geo-statistical approach. *Nature Communications*. 2016; **7**(1): 13603. doi: 10.1038/ncomms13603

4.	Feng L, Dai Y, Hou X *et al.* Concerns about phytoplankton bloom trends in global lakes. *Nature*. 2021; **590**(7846): E35-E47. doi: 10.1038/s41586-021-03254-3

5.	Liu C, Zhu L, Wang J *et al.* In-situ water quality investigation of the lakes on the Tibetan Plateau. *Science Bulletin*. 2021; **66**(17): 1727-1730. doi: <https://doi.org/10.1016/j.scib.2021.04.024>

6.	Wen Z, Song K, Lyu L *et al.* A national-scale data set for dissolved carbon and its spatial pattern in lakes and reservoirs across China. *Scientific Data*. 2020; **7**(1): 82. doi: 10.1038/s41597-020-0419-5

7.	Boegehold AG, Burtner AM, Camilleri AC *et al.* Routine monitoring of western Lake Erie to track water quality changes associated with cyanobacterial harmful algal blooms. *Earth Syst Sci Data*. 2023; **15**(8): 3853-3868. doi: 10.5194/essd-15-3853-2023

8.	Drayson N, Anstee J, Botha H *et al.* Australian aquatic bio-optical dataset with applications for satellite calibration, algorithm development and validation. *Data in Brief*. 2022; **44**: 108489. doi: <https://doi.org/10.1016/j.dib.2022.108489>

9.	Lehmann MK, Gurlin D, Pahlevan N *et al.* GLORIA - A globally representative hyperspectral in situ dataset for optical sensing of water quality. *Scientific Data*. 2023; **10**(1): 100. doi: 10.1038/s41597-023-01973-y

10.	IMF. *World economic outlook*. Washington, DC: International Monetary Fund; 2023. (World economic outlook) (DHHS publication no.: Report Number)| (GPO o. Document Number)|.

11.	UNCTAD. *The Least Developed Countries Report*. Geneva: UN Trade and Development; 2023. (The Least Developed Countries Report) (DHHS publication no.: Report Number)| (GPO o. Document Number)|.

12.	Zhong D, Lin Y, Yu J *et al.* Long-term algal blooms mapping and crucial driving factors analysis based on Landsat series imagery in Lake Victoria (2001–2021). *ISPRS Ann Photogramm Remote Sens Spatial Inf Sci*. 2025; **X-G-2025**: 1061-1068. doi: 10.5194/isprs-annals-X-G-2025-1061-2025

13.	Lloyd CT, Sorichetta A, Tatem AJ. High resolution global gridded data for use in population studies. *Scientific Data*. 2017; **4**(1): 170001. doi: 10.1038/sdata.2017.1

14.	Lloyd CT, Chamberlain H, Kerr D *et al.* Global spatio-temporally harmonised datasets for producing high-resolution gridded population distribution datasets. *Big earth data*. 2019; **3**(2): 108-139.

15.	Cao Z, Ma R, Liu J *et al.* Improved Radiometric and Spatial Capabilities of the Coastal Zone Imager Onboard Chinese HY-1C Satellite for Inland Lakes. *IEEE Geoscience and Remote Sensing Letters*. 2020: 1-5. doi: 10.1109/LGRS.2020.2971629

16.	Hu C, Feng L, Lee Z *et al.* Dynamic range and sensitivity requirements of satellite ocean color sensors: learning from the past. *Applied Optics*. 2012; **51**(25): 6045-6062. doi: 10.1364/AO.51.006045

17.	Carrea L, Crétaux J-F, Liu X *et al.* ESA Lakes Climate Change Initiative (Lakes_cci): Lake products, Version 2.0.2. Didcot, UK: NERC EDS Centre for Environmental Data Analysis; 2022.

18.	Hu C. A novel ocean color index to detect floating algae in the global oceans. *Remote Sensing of Environment*. 2009; **113**(10): 2118-2129.

19.	Hou X, Feng L, Duan H *et al.* Fifteen-year monitoring of the turbidity dynamics in large lakes and reservoirs in the middle and lower basin of the Yangtze River, China. *Remote Sensing of Environment*. 2017; **190**: 107-121. doi: 10.1016/j.rse.2016.12.006

20.	Liu G, Li S, Song K *et al.* Remote sensing of CDOM and DOC in alpine lakes across the Qinghai-Tibet Plateau using Sentinel-2A imagery data. *Journal of Environmental Management*. 2021; **286**: 112231. doi: <https://doi.org/10.1016/j.jenvman.2021.112231>

21.	Maciel DA, Barbosa CCF, Novo EMLdM *et al.* Water clarity in Brazilian water assessed using Sentinel-2 and machine learning methods. *ISPRS Journal of Photogrammetry and Remote Sensing*. 2021; **182**: 134-152. doi: <https://doi.org/10.1016/j.isprsjprs.2021.10.009>

22.	Wang M, Shi W. The NIR-SWIR combined atmospheric correction approach for MODIS ocean color data processing. *Optics Express*. 2007; **15**(24): 15722-15733. doi: 10.1364/OE.15.015722

23.	Wang S, Li J, Zhang B *et al.* Changes of water clarity in large lakes and reservoirs across China observed from long-term MODIS. *Remote Sensing of Environment*. 2020; **247**: 111949. doi: <https://doi.org/10.1016/j.rse.2020.111949>

24.	MEE. Environmental quality standards for surface water (GB 3838-2002). Beijing: Ministry of Ecology and Environment; 2002.

25.	UN-Water. *Integrated Monitoring Guide for SDG 6 - Targets and global indicators*. New York: UN-Water; 2016 (DHHS publication no.: Report Number)| (GPO o. Document Number)|.

26.	USEPA. Water Quality Standards. Washington, D.C.2013.

27.	EU. Directive 2000/60/EC of the European Parliament and of the Council of 23 October 2000 establishing a framework for Community action in the field of water policy. Brussels: Office for Official Publications of the European Communities; 2000.

28.	Moeinzadeh H, Yong K-T, Withana A. A critical analysis of parameter choices in water quality assessment. *Water Research*. 2024; **258**: 121777. doi: <https://doi.org/10.1016/j.watres.2024.121777>

29.	Kumar D, Kumar R, Sharma M *et al.* Global water quality indices: Development, implications, and limitations. *Total Environment Advances*. 2024; **9**: 200095. doi: <https://doi.org/10.1016/j.teadva.2023.200095>

30.	Shen M, Duan H, Cao Z *et al.* Sentinel-3 OLCI observations of water clarity in large lakes in eastern China: Implications for SDG 6.3.2 evaluation. *Remote Sensing of Environment*. 2020; **247**: 111950. doi: <https://doi.org/10.1016/j.rse.2020.111950>

31.	Breiman L. Random forests. *Machine learning*. 2001; **45**(1): 5-32.

32.	Bergstra J, Yamins D, Cox D. Making a science of model search: Hyperparameter optimization in hundreds of dimensions for vision architectures. In: *International conference on machine learning,* *2013*, p. 115-123. PMLR.

33.	Gurlin D, Gitelson AA, Moses WJ. Remote estimation of chl-a concentration in turbid productive waters — Return to a simple two-band NIR-red model? *Remote Sensing of Environment*. 2011; **115**(12): 3479-3490. doi: <https://doi.org/10.1016/j.rse.2011.08.011>

34.	O'Reilly JE, Maritorena S, Mitchell BG *et al.* Ocean color chlorophyll algorithms for SeaWiFS. *Journal of Geophysical Research Atmospheres*. 1998; **103**(C11): 24937-24950.

35.	Smith ME, Robertson Lain L, Bernard S. An optimized Chlorophyll a switching algorithm for MERIS and OLCI in phytoplankton-dominated waters. *Remote Sensing of Environment*. 2018; **215**: 217-227. doi: <https://doi.org/10.1016/j.rse.2018.06.002>

36.	Gons HJ, Rijkeboer M, Ruddick KG. A chlorophyll-retrieval algorithm for satellite imagery (Medium Resolution Imaging Spectrometer) of inland and coastal waters. *Journal of Plankton Research*. 2002; **24**(9): 947-951. doi: 10.1093/plankt/24.9.947

37.	Miller RL, McKee BA. Using MODIS Terra 250 m imagery to map concentrations of total suspended matter in coastal waters. *Remote Sensing of Environment*. 2004; **93**(1): 259-266. doi: <https://doi.org/10.1016/j.rse.2004.07.012>

38.	Xue K, Ma R, Duan H *et al.* Inversion of inherent optical properties in optically complex waters using sentinel-3A/OLCI images: A case study using China's three largest freshwater lakes. *Remote Sensing of Environment*. 2019; **225**: 328-346. doi: 10.1016/j.rse.2019.03.006

39.	Yu X, Lee Z, Shen F *et al.* An empirical algorithm to seamlessly retrieve the concentration of suspended particulate matter from water color across ocean to turbid river mouths. *Remote Sensing of Environment*. 2019; **235**: 111491. doi: <https://doi.org/10.1016/j.rse.2019.111491>

40.	Nechad B, Ruddick KG, Park Y. Calibration and validation of a generic multisensor algorithm for mapping of total suspended matter in turbid waters. *Remote Sensing of Environment*. 2010; **114**(4): 854-866. doi: <https://doi.org/10.1016/j.rse.2009.11.022>

41.	Kutser T, Pierson DC, Kallio KY *et al.* Mapping lake CDOM by satellite remote sensing. *Remote Sensing of Environment*. 2005; **94**(4): 535-540.

42.	Mannino A, Russ ME, Hooker SB. Algorithm development and validation for satellite-derived distributions of DOC and CDOM in the U.S. Middle Atlantic Bight. *Journal of Geophysical Research: Oceans*. 2008; **113**(C7). doi: <https://doi.org/10.1029/2007JC004493>

43.	Zhu W, Yu Q, Tian YQ *et al.* An assessment of remote sensing algorithms for colored dissolved organic matter in complex freshwater environments. *Remote Sensing of Environment*. 2014; **140**: 766-778. doi: 10.1016/j.rse.2013.10.015

44.	Shi K, Zhang Y, Zhu G *et al.* Deteriorating water clarity in shallow waters: Evidence from long term MODIS and in-situ observations. *International Journal of Applied Earth Observation and Geoinformation*. 2018; **68**: 287-297. doi: 10.1016/jjag.2017.12.015

45.	Caballero I, Stumpf RP, Meredith A. Preliminary Assessment of Turbidity and Chlorophyll Impact on Bathymetry Derived from Sentinel-2A and Sentinel-3A Satellites in South Florida. *Remote Sensing* 2019, doi: 10.3390/rs11060645

46.	Ren J, Zheng Z, Li Y *et al.* Remote observation of water clarity patterns in Three Gorges Reservoir and Dongting Lake of China and their probable linkage to the Three Gorges Dam based on Landsat 8 imagery. *Science of The Total Environment*. 2018; **625**: 1554-1566. doi: 10.1016/j.scitotenv.2018.01.036

47.	Alikas K, Kratzer S. Improved retrieval of Secchi depth for optically-complex waters using remote sensing data. *Ecological Indicators*. 2017; **77**: 218-227.

48.	Lee Z, Shang S, Qi L *et al.* A semi-analytical scheme to estimate Secchi-disk depth from Landsat-8 measurements. *Remote Sensing of Environment*. 2016; **177**: 101-106. doi: 10.1016/j.rse.2016.02.033

49.	Guan Q, Feng L, Hou X *et al.* Eutrophication changes in fifty large lakes on the Yangtze Plain of China derived from MERIS and OLCI observations. *Remote Sensing of Environment*. 2020; **246**: 111890. doi: <https://doi.org/10.1016/j.rse.2020.111890>

50.	Cao Z, Ma R, Duan H *et al.* A machine learning approach to estimate chlorophyll-a from Landsat-8 measurements in inland lakes. *Remote Sensing of Environment*. 2020; **248**: 111974. doi: <https://doi.org/10.1016/j.rse.2020.111974>

51.	UNEP. *Global indicator 6.3.2 updates and acceleration needs*. New York: UN Environment Programme on behalf of UN-Water; 2021. (Progress on Ambient Water Quality) (DHHS publication no.: Report Number)| (GPO o. Document Number)|.

52.	Pahlevan N, Greb S, Dekker AG. Earth Observation in Support of SDG 6.3.2/6.6.1. *Earth Observation Applications and Global Policy Frameworks*2022. 67-83.

53.	Shen M, Cao Z, Xie L *et al.* Microcystins risk assessment in lakes from space: Implications for SDG 6.1 evaluation. *Water Research*. 2023; **245**: 120648. doi: <https://doi.org/10.1016/j.watres.2023.120648>

54.	Servos MR, Munkittrick KR, Constantin G *et al.* Science and management of transboundary lakes: Lessons learned from the global environment facility program. *Environmental Development*. 2013; **7**: 17-31. doi: <https://doi.org/10.1016/j.envdev.2013.04.005>

55.	Stålnacke P, Roll G. Lake Peipsi: A transboundary lake on the future border of the European Union. *The NEBI YEARBOOK 2001/2002: North European and Baltic Sea Integration*: Springer; 2002. 159-176.

56.	Tammeorg O, Tuvikene L, Kondratyev S *et al.* Opportunities for combining data of Estonian and Russian monitoring to reflect on water quality in large transboundary Lake Peipsi. *Journal of Great Lakes Research*. 2022; **48**(4): 961-970. doi: <https://doi.org/10.1016/j.jglr.2022.05.009>

57.	UNEP. *Piloting the monitoring methodology and initial fndings for SDG indicator 6.3.2*. New York: UN Environment on behalf of UN-Water; 2018. (Progress on Ambient Water Quality) (DHHS publication no.: Report Number)| (GPO o. Document Number)|.

58.	Zhi W, Appling AP, Golden HE *et al.* Deep learning for water quality. *Nature Water*. 2024; **2**(3): 228-241. doi: 10.1038/s44221-024-00202-z
